# Proximity Labeling Techniques to Study Chromatin

**DOI:** 10.3389/fgene.2020.00450

**Published:** 2020-05-12

**Authors:** Henning Ummethum, Stephan Hamperl

**Affiliations:** Chromosome Dynamics and Genome Stability, Institute of Epigenetics and Stem Cells, Helmholtz Zentrum München, Munich, Germany

**Keywords:** protein-protein interactions, proxisome, BioID, APEX2, dCas9, ChIP, affinity purification, mass spectrometry

## Abstract

Mammals contain over 200 different cell types, yet nearly all have the same genomic DNA sequence. It is a key question in biology how the genetic instructions in DNA are selectively interpreted by cells to specify various transcriptional programs and therefore cellular identity. The structural and functional organization of chromatin governs the transcriptional state of individual genes. To understand how genomic loci adopt different levels of gene expression, it is critical to characterize all local chromatin factors as well as long-range interactions in the 3D nuclear compartment. Much of our current knowledge regarding protein interactions in a chromatin context is based on affinity purification of chromatin components coupled to mass spectrometry (AP-MS). AP-MS has been invaluable to map strong protein-protein interactions in the nucleus. However, the interaction is detected after cell lysis and biochemical enrichment, allowing for loss or gain of false positive or negative interaction partners. Recently, proximity-dependent labeling methods have emerged as powerful tools for studying chromatin in its native context. These methods take advantage of engineered enzymes that are fused to a chromatin factor of interest and can directly label all factors in proximity. Subsequent pull-down assays followed by mass spectrometry or sequencing approaches provide a comprehensive snapshot of the proximal chromatin interactome. By combining this method with dCas9, this approach can also be extended to study chromatin at specific genomic loci. Here, we review and compare current proximity-labeling approaches available for studying chromatin, with a particular focus on new emerging technologies that can provide important insights into the transcriptional and chromatin interaction networks essential for cellular identity.

## Introduction

A long-standing question in cell biology is how the same genome can lead to different cell types. A major driving force that determines cellular identity is their underlying gene expression landscape. Whether a gene is turned on or off depends mostly on its physical accessibility, governed by the local chromatin context (Klemm et al., 2019). The basic unit of chromatin is the nucleosome core particle, a protein-DNA complex consisting of 146 bp of DNA wrapped around a histone octamer. Histones play a central role in DNA accessibility, due to histone variants and a multitude of post-translational modifications (PTMs) that influence binding of secondary chromatin factors. This can lead to further compaction and heterochromatin formation, restricting or completely blocking access for the transcription machinery. Other factors influencing chromatin structure are DNA methylation, long non-coding RNAs and chromatin remodelers. However, the full extent of chromatin modifications and complex interactions of a given gene in the complex nuclear environment are poorly understood. Therefore, it is crucial to identify all factors that are part of this process by studying protein-protein interactions of known chromatin factors.

The most widely applied methods to study protein-protein interactions in a chromatin context are affinity purification or immunoprecipitation followed by mass spectrometry (AP-MS/IP-MS). After cell lysis, soluble proteins are captured and enriched by a ligand (bait) coupled to a solid support. The most commonly used ligands are antibodies targeting epitope-tagged (AP) or endogenous (IP) proteins (prey). These ligands are attached to a solid support, in most cases agarose, sepharose or magnetic beads. After enrichment, proteins are analyzed by mass spectrometry to identify proteins interacting with the prey. Consequently, successful application of IP-MS depends on the availability of an antibody or known interactor of the protein of interest. For AP-MS, common antibodies can be used, because an epitope tag is fused to the protein of interest. While these methods can efficiently identify strong protein-protein interactions that are not disrupted after cell lysis and solubilization, transient interactors with lower affinity can be lost during the purification steps – typically performed under high-salt and detergent conditions. In addition, interactions are only detected after lysis and enrichment and thus no longer in its native environment of living cells.

Proximity labeling followed by mass spectrometry analysis can address these key limitations of AP- and IP-MS. The basic principle of all proximity labeling methods is to introduce a covalent biotin tag to proteins in the neighborhood of a selected target in living cells. To this end, enzymes convert a supplemented substrate into a highly reactive biotinylated intermediate that then transfers biotin to amino acid side chains in proximity. Spatial restriction of labeling is achieved by fusing the enzyme to the target protein as well as reducing the labeling time. Currently, three major enzymes are used for proximity labeling: biotin ligase (BioID, BioID2, TurboID, miniTurbo), horse radish peroxidase (HRP), and engineered ascorbate peroxidase (APEX, APEX2). After the labeling reaction, cells are lysed and the biotinylated proteins are extracted with streptavidin beads and subjected to mass spectrometry. Identified candidates in proximity with the bait protein can be summarized as the “proxisome” (Roux et al., 2012).

The advantages of proximity labeling in comparison to conventional methods to study chromatin are manifold. One major benefit is the ability to analyze protein interactions in a native context, because covalent biotinylation occurs before cell lysis and solubilization. As streptavidin-biotin is one of the strongest non-covalent interactions found in nature, harsh conditions can be used to force insoluble proteins into solution without the constraint of maintaining protein-protein interactions during the purification process. Therefore, proximity labeling enables the study of proxisomes even in insoluble cell compartments like the nuclear matrix, nucleoli and other nuclear structures – difficult to study with conventional methods. Additionally, *in vivo* covalent biotinylation enables the detection of transient interactions and low abundance proteins. Finally, biotinylation is an infrequent protein modification in many organisms, thus no additional endogenous proteins are part of the background in mass spectrometry analysis (de Boer et al., 2003).

Here, we will review and compare current proximity labeling approaches available for studying chromatin, with a particular focus on new emerging technologies that can provide important insights into the transcriptional and chromatin interaction networks from specific gene loci to whole genome interactions in nuclear compartments.

## Proximity Labeling Methods

### Biotin Ligase (BioID)

The *Escherichia coli* BirA biotin ligase converts biotin and ATP into biotinoyl-5′-adenylate (bioAMP) (Barker and Campbell, 1981a, b; Eisenberg et al., 1982). One of the physiological roles of the BirA-bioAMP complex is to target the only biotinylation site in *E. coli*, a lysine residue in the biotin carboxyl carrier protein (BCCP) subunit of acetyl-CoA carboxylase. To take advantage of this highly specific reaction, an unnatural substrate mimicking a short peptide sequence was created (Schatz, 1993; Beckett et al., 1999). This biotin acceptor peptide (BAP) can be fused to proteins of interest (POI) and co-expressed with BirA, which in turn recognizes and conjugates biotin on the lysine of BAP (Smith et al., 1998). The newly biotinylated protein can be efficiently purified by streptavidin pull-down (de Boer et al., 2003). In a different approach, this system was used to study protein-protein interactions by fusing BirA and BAP to two interacting proteins (Fernández-Suárez et al., 2008). However, interacting protein pairs must be known *a priori*.

**FIGURE 1:**
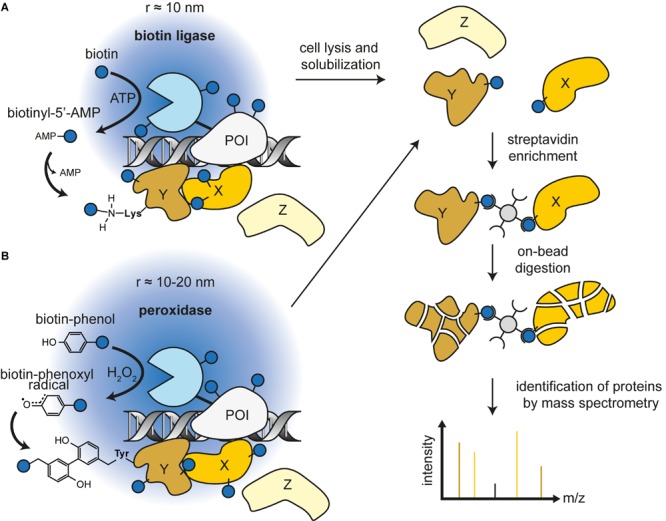
General workflow of proximity labeling followed by mass spectrometry with biotin ligase **(A)** or peroxidase **(B)**. The protein of interest (bait) is fused to the reporter enzyme and expressed in cells. Supplying the enzymes with their substrates creates reactive intermediates that target amino acid side chains of proteins in proximity (prey). The covalently biotinylated proteins can be enriched by streptavidin beads. Subsequent on-bead digestion and identification of resulting peptides with mass spectrometry provides a candidate list of proteins in the vicinity of the bait.

A mutated BirA^∗^ (R118G) from *E. coli* made an unbiased approach possible by disrupting binding of bioAMP to BirA (Kwon and Beckett, 2000; Kwon et al., 2000). Consequently, bioAMP diffuses from the enzyme and can readily react with lysine residues of any protein. Interestingly, *in vitro* experiments showed that biotinylation efficiency is proximity-dependent, meaning that substrates closer to BirA^∗^ were more readily biotinylated (Choi-Rhee et al., 2004; Cronan, 2005). To promiscuously biotinylate proteins in mammalian cells, a codon-optimized BirA^∗^ was designed and fused to the protein of interest (Roux et al., 2012). With this approach, termed BioID, it was now possible to identify the proximal proteome of in theory any protein of interest. By switching from the *E. coli* to the *Aquifex aeolicus* biotin ligase, the size of the BioID moiety was reduced from 35 to 28 kDa (Kim et al., 2016). Later, it was possible to reduce the labeling time from a minimum of 6 h to 10 min with an *E. coli* biotin ligase mutated at 14 amino acids, namely TurboID (Branon et al., 2018). In parallel, a mutated and truncated biotin ligase from *Bacillus subtilis* (BASU) was developed and achieved efficient labeling for subsequent LC-MS/MS analysis in 30 min (Ramanathan et al., 2018). However, this improved activity was only demonstrated in a very specific context in which BirA^∗^ is fused to a small peptide that recognizes RNA motifs. Furthermore, during the development of TurboID/miniTurboID, BASU showed kinetics comparable with BioID and BioID2 (Branon et al., 2018; Figure 1A; and Table 1).

**TABLE 1:** Overview of available proximity labeling enzymes and their characteristics.

Enzyme	Type	Source organism	Ami no acid mutations	Size in kDa	Labeling time	Substrate incubation time	Substrates	Labeling targets	Reference
BiolD	Biotin ligase	*E. coli*	R118G	35	6–24 h	6–24 h	Biotin	Lys	Roux et al., 2012
BiolD2	Biotin ligase	*A. aeolicus*	R40G	27	6–24 h	6–24 h	Biotin	Lys	Kim et al., 2016
BASU	Biotin ligase	*B. subtilis*	13 mut., ΔN-term	29	30 min-12 h	30 min-12 h	Biotin	Lys	Ramanathan et al., 2018
miniTurbo	Biotin ligase	*E. coli*	12 mut., ΔN-term	28	10–60 min	10–60 min	Biotin	Lys	Branon et al., 2018
TurbolD	Biotin ligase	*E. coli*	14 mut., ΔN-term	35	10–60 min	10–60 min	Biotin	Lys	Branon et al., 2018
HRP	Peroxidase	Horseradish	–	44	5–10 min	5–10 min	Biotin-phenol, Fluorescein-aryl azide	Tyr, Trp, Cys, His	Kotani et al., 2008; Li et al., 2014; Rees et al., 2015
APEX	Peroxidase	Pea	K14D, E112K, W41F	28	1 min	30–60 min	Biotin-phenol	Tyr, Trp, Cys, His	Martell et al., 2012; Rhee et al., 2013
APEX2	Peroxidase	Soybean	K14D, E112K, W41F, A134P	28	1 min	30–60 min	Biotin-phenol, -aniline, -naphthylamine	Tyr, Trp, Cys, His	Lam et al., 2015

### Engineered Ascorbate Peroxidase (APEX)

Peroxidases can oxidize various chromogenic substrates in the presence of H_2_O_2_, making it a versatile tool for biochemistry applications. For example, horseradish peroxidase (HRP) has been used to enhance contrast for electron microscopy by polymerizing 3,3′-diaminobenzidine after OsO_4_ treatment (Graham and Karnovsky, 1966; Li et al., 2010). Peroxidases can also catalyze the oxidation of phenol derivatives to phenoxyl radicals (Gross and Sizer, 1959). This chemistry is the basis of tyramide signal amplification, a widely used technique for immunostainings (Mayer and Bendayan, 1997). Phenoxyl radicals can also react with electron-rich amino acids, predominantly tyrosine (>95%), but also tryptophan and cysteine (Udeshi et al., 2017). Because these radicals are very short lived (<1 ms), they can only react with amino acid residues in close proximity of the peroxidase (Mortensen and Skibsted, 1997). The first biotin-based proximity labeling study was done with HRP and aryl azide-biotin as substrate (Kotani et al., 2008). However, HRP is not active in the mammalian cytosol, because two essential disulfide bridges cannot form in the reducing environment (Martell et al., 2012). Introduction of an engineered ascorbate peroxidase (APEX) derived from pea overcame this caveat (Rhee et al., 2013). It is active in all cell compartments and can label surrounding proteins through incubation with H_2_O_2_ and biotin-phenol. The biotin-phenoxyl radicals primarily target tyrosine residues at surface-exposed sites of proteins. Furthermore, APEX with 28 kDa has a lower molecular weight opposed to the 44 kDa HRP, making the fusion protein less likely to compromise the native structure or function of the protein. The low catalytic activity of this first APEX version prompted a directed evolution approach and the development of the more active A134P mutated version of the enzyme named APEX2 (Lam et al., 2015; Figure 1B; and Table 1).

### General Considerations for BioID and APEX Experiments

In summary, biotin ligases and ascorbate peroxidases provide a powerful tool to investigate the proximity of a protein of interest, giving insight into potential interaction partners. Nevertheless, fusing a relatively large 27–28 kDa enzyme to the bait protein may influence its function and/or localization (Roux et al., 2012; Roux, 2013; Kim and Roux, 2016). The moiety has a similar size as other common tags, e.g., green fluorescence protein (GFP). Consequently, a good practice might be to fuse the proximity labeling enzyme to N- or C-termini of target proteins that have already been successfully tagged with GFP or another moiety in the same size range. In general, the concept of proximity labeling does not allow direct testing for interaction partners, but rather provide a candidate list of possible interactors (Roux et al., 2012). The functional relevance of these candidates should then be validated by further experimentation.

Additionally, the labeling radius is not clear, especially for biotin ligases. The reactive bioAMP has a half-life of minutes, potentially enabling it to diffuse away from the biotin ligase (Rhee et al., 2013). However, a BioID study of the nuclear pore complex reported an effective labeling radius of only ∼10 nm (Kim et al., 2014). Interestingly, the insertion of a flexible linker into the fusion protein can increase the labeling radius (Kim et al., 2016). The APEX2 generated biotin-phenoxyl radicals are very short lived (<1 ms), which leads to a decreasing degree of biotinylation with increasing physical radius from the peroxidase (Hung et al., 2016). When combining APEX2 biotinylation with the ratiometric Stable Isotope Labeling with Amino Acids in Cell Culture (SILAC) approach, it is possible to achieve high spatial resolution, especially in non-membrane enclosed compartments (Hung et al., 2016). Electron microscopy images suggest the labeling radius of biotin-phenoxyl radicals to be ∼10–20 nm (Mayer and Bendayan, 1997). Another drawback is that the strong biotin-streptavidin bond does not allow for efficient elution of biotinylated proteins from the beads. This is usually circumvented by on-bead digestion, but interactions of non-biotinylated proteins with the beads can introduce many false positives. Additionally, the biotinylated peptides that are cleaved off the beads still containing part of streptavidin are too complex to be analyzed by mass spectrometry, leading to a loss of important peptides for later analysis. New methods, e.g., Biotin Site Identification Technology (BioSITe) and Direct Detection of Biotin-containing Tags (DiDBit) aim at addressing these issues by first digesting the proteins and subsequently enriching with biotin nano- or antibodies (Schiapparelli et al., 2014; Kim et al., 2018). Using antibodies does not lead to complex undetectable peptides. Also, this approach can potentially increase sensitivity, because enrichment on the peptide level greatly reduces the background of non-biotinylated peptides (Udeshi et al., 2017; Kim et al., 2018). Additionally, this approach allowed the identification of the preferential biotinylation sites on proteins (Udeshi et al., 2017).

When designing a proximity labeling experiment, an important point to consider is that large amounts of false positives can be generated due to random spatial association with the protein of interest. Consequently, negative controls are mandatory and should always be included in the experimental setup (Lobingier et al., 2017). In general, two types of controls are recommended – a technical control without the proximity labeling reaction and importantly, a spatial control mimicking the reaction at specific subcellular locations. Technical controls give insight into contaminants arising through the enrichment strategy, whereas the spatial control expresses the enzyme alone or fused to a localization tag, e.g., NLS-BirA^∗^, and provides information of common contaminants of the labeling reaction itself. Furthermore, it is crucial to limit and achieve similar expression levels of bait and control fusion proteins, otherwise different levels of background can mask bona fide interactions. For BioID, cells with no BirA^∗^, BirA^∗^ alone, or BirA^∗^ fused to a localization tag are the three most common controls. This is transferrable to APEX experiments, but it is also possible to omit H_2_O_2_ or biotin phenol instead of using no APEX. Furthermore, a database named CRAPome for known contaminants in immunoprecipitation and BioID experiments has been established (Mellacheruvu et al., 2013). It is possible to select specific negative controls from other studies, e.g., NLS-BirA^∗^ if the experimental designs are highly similar. However, if the cell type or the enrichment strategy of the control differs significantly, it is always recommended to include an internal experimental control, rather than solely relying on the CRAPome database. Furthermore, experimental design also entails whether to use a qualitative or one of the many quantitative mass spectrometry approaches. There does not seem to be a preferential method for proximity labeling, so it comes down to technical considerations (see Santin, 2019 for details and Ankney et al., 2016 for a comprehensive summary of quantitative approaches).

Another point of consideration for proximity labeling is that the amount of biotinylation does not necessarily reflect the strength of association. In fact, biotinylation relies on the number and accessibility of the targeted amino acid residues, mostly lysine or tyrosine. This also means that intrinsically disordered regions of proteins, which are very sensitive to changes in pH, salt concentration and PTMs, can introduce biases in proximity labeling studies (Minde et al., 2020). On average, the preferentially targeted lysines in BioID are a lot more abundant in intrinsically disordered regions than tyrosines preferred by APEX. This could also explain the fact that a biotinylation gradient is observed with APEX, but not with BioID.

## Chromatin Factors Targeted by Proximity Labeling

**FIGURE 2:**
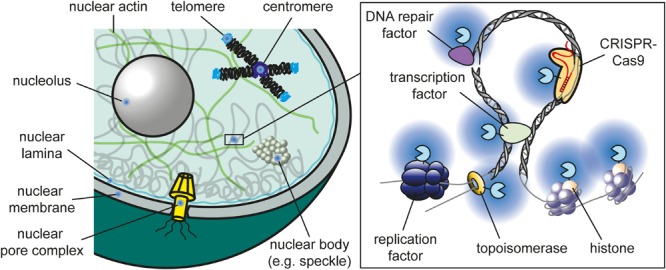
Nuclear compartments that were successfully targeted by proximity labeling. A detailed compiled list of studies is provided in Supplementary Table S1.

Proximity labeling has been used to study chromatin factors in many different nuclear compartments (Figure 2). In the following sections, selected studies are presented covering a wide range of proximity labeling techniques. A more complete list can be found in Supplementary Table S1.

### Histone Variants and Post-translational Modifications

One of the first approaches to study chromatin with biotin ligase was to identify histone modifications in the vicinity of RAD18 (Shoaib et al., 2013). The authors fused BirA to RAD18 and BAP to histones H3.1 and H2A. By combining this approach with native Chromatin Immunoprecipitation (NChIP), it was found that the H4 histones in proximity to RAD18 are hyperacetylated compared to bulk histones. Importantly, this study proved the feasibility to fuse BirA to any nuclear protein of interest and determine features of the surrounding histones. However, the extensive MNase digestion only creates biotinylated mononucleosomes, therefore excluding the analysis of non-histone protein interactions.

The development of BioID has overcome this limitation. Additionally, BioID applications are technically less challenging, as only one genetic fusion of BirA^∗^ to the protein of interest is required without the counterpart BAP. By fusing BirA^∗^ to the histone H3 like nucleoprotein CENP-A, HJURP was identified as a centromere associating protein in S phase (Zasadzińska et al., 2018). This interaction was later confirmed by *in vitro* BioID (ivBioID) (Remnant et al., 2019). In this variation of the assay, the biotin substrate is only added after a brief pre-extraction period and therefore allows quick substrate penetration and biotinylation in a timescale of minutes. This addresses the shortcomings of the regular BioID approach, which needs a biotin incubation time of at least 6 h. However, it is less suited for soluble proteins, because they are washed from the cells after permeabilization. Furthermore, it does not require treatment of cells with H_2_O_2_, a potentially oxidative damage-inducing agent. However, the use of H_2_O_2_ in the regular APEX2 protocol at low concentrations and short time periods of 60 s may not severely impact signaling pathways (Veal et al., 2007). Also, the development of TurboID reduced the biotin labeling time to 10 min, addressing the same issue of the standard BioID. Nevertheless, ivBioID seems to provide lower background and can resolve even finer time intervals, providing a snapshot of the proxisome at the time of lysis. In addition, ivBioID can be used in any genetically modifiable organism regardless of difficulties with endogenous biotin levels or biotin delivery.

In contrast, the APEX2 approach has been developed mainly in mammalian cells and is not easily transferable to other organisms. The main concern is the delivery of the substrate biotin phenol into the cell or nucleus (Hung et al., 2016). For yeast, removing the cell wall by zymolyase or osmotic shock allows the entry of biotin phenol (Hwang and Espenshade, 2016). Further optimization of the protocol by a different group enabled the proteomic mapping of the mitochondrial matrix and the nucleus (Singer-Krüger et al., 2019). As an example, fusion of APEX2 to the core H2B histone Htb1 identified Yer156c, a nuclear protein with unknown function previously not detected with traditional IP-MS approaches.

Recently, a method named ChromID to study the proxisome of specific histone PTMs was published (Villaseñor et al., 2020). In this approach, engineered chromatin readers (eCRs) are fused to the biotin ligase BASU. In this study, eCRs for histone tri-methylated H3K4, H3K9, and H3K27 have been developed and successfully used with proximity labeling. Additionally, the authors were able to employ a bivalent eCR to study the proxisome at H3K4me3 and H3K27me3 marked sites. This method has very promising potential for studying associating factors of histone modifications in different conditions. It might also be useful for tracking histone mark proxisomes during developmental changes. However, with a labeling time of 12 h, ChromID might be less suitable to study dynamic cellular processes.

### Transcription Factors

**FIGURE 3:**
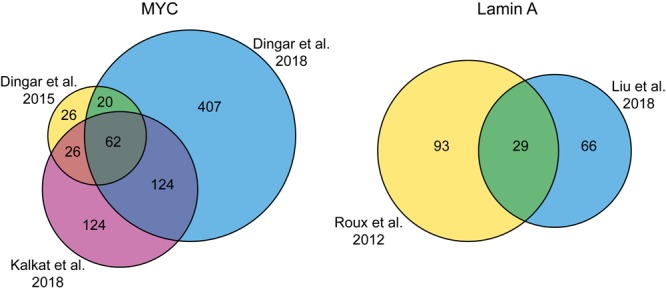
Venn diagrams showing the number of shared identifications in BioID studies with the same bait. **(A)** Comparison of three studies with the MYC protein as bait. **(B)** Comparison of two studies with Lamin A as bait.

Multiple proxisomes of transcription factors have been uncovered with the help of proximity labeling. Fusion of BirA^∗^ to the MYC oncoprotein in cultured HEK293 and tumor xenografts confirmed known and identified over 70 new potential interaction partners, ranging from chromatin remodelers to transcription factors (Dingar et al., 2015). Therefore, proximity labeling significantly improved our knowledge of potential MYC interactors, which has been difficult to study with classical IP/AP-MS due to difficult to solubilize chromatin-bound complexes containing MYC. Later, the same group identified protein phosphatase 1 (PP1) and its regulatory subunit protein phosphatase-1 nuclear-targeting subunit (PNUTS) as MYC-interactors in HeLa cells (Dingar et al., 2018). In an additional study, the six highly conserved MYC homology boxes (MBs) were individually deleted and the mutants were fused to BirA^∗^ in HEK293 cells (Kalkat et al., 2018). Some of these MBs are crucial for MYC-dependent malignant transformation. The resulting six BioID proxisomes were compared to the wild type proxisome and gave important insights into the binding targets of the individual MYC homology boxes. Interestingly, when comparing these three MYC proxisome studies done in HeLa cells and HEK293 cells/tumor xenografts, a large overlap of 62 candidates can be observed despite the disparity of cellular systems (Figure 3). This suggests that BioID can efficiently and reproducibly detect specific interactions and these can be considered as the “core” high-confidence hits, whereas the other candidates only detected in one or two of the studies may contain more bona fide targets in a rather cell- and context-specific manner. In general, this example also illustrates that proximity labeling techniques can potentially discriminate between such a hierarchy of different interaction levels.

GFI1B is a master regulator of developmental hematopoiesis, which can also play both oncogenic and oncosuppressor roles in hematologic malignancies (Anguita et al., 2017). To study GFI1B’s proxisome, a GFI1B-BioID2 fusion protein was used (McClellan et al., 2019). Besides many known interactors and members of other transcription complexes, the H3K4me1/2 and H3K9me1/2 specific lysine demethylase LSD1 was identified. To repress transcription, GFI1B needs to bind to LSD1 via its SNAG domain. To identify LSD1-dependent transcriptional regulatory complexes, BioID2 fusions with a wildtype SNAG domain or different mutant alleles were created. Importantly, specific enrichment of the BRAF-HDAC complex (BHC) was only detected in the proxisome of the constructs with intact SNAG domain. Consequently, proximity labeling was able to identify LSD1 dependence of the BHC complex. Thus, the two examples of GFI1B and MYC show that combining proximity labeling data sets of different deletion mutants of the same protein can even identify the interactome of specific functional domains.

Another transcription factor fused to BirA^∗^ is SOX2 (Kim et al., 2017). Copy number gains of SOX2 arise in almost all squamous cell carcinomas (SQCC) of the lung, suggesting a functional role in disease progression (Weina and Utikal, 2014). Similar to many other transcription factors, direct targeting of SOX2 by small molecule inhibitors was not successful. The first SOX2 proxisome analysis by BioID confirmed the association with histone acetyltransferase EP300 in HEK293 cells, an interaction that was not clear due to conflicting AP-MS studies (Kim et al., 2017). This approach illustrates how proximity labeling can be used to screen for interaction partners of “non-druggable” oncoproteins that can then be targeted for improved therapeutic control of transcription factor oncogenic functions. Interestingly, of the 82 candidates, 46 were also found in at least one of eight AP-MS SOX2 interactome studies, suggesting BioID is able to identify new and verify many known interactions.

The fusion protein EWS-Fli-1 can be generated after chromosomal translocations and is present in most cases of Ewing sarcoma, an aggressive form of bone cancer (Li et al., 2015). To assess the interactome of EWS-Fli-1, a tandem affinity purification approach was first applied (Elzi et al., 2014). However, the majority of the expressed and tagged fusion protein was not effectively solubilized under non-denaturing conditions. Again, this limitation was overcome by BioID, where they could detect and subsequently verify a connection between the lysosome and EWS-Fli-1 protein turnover. Interestingly, this could be achieved with ∼10 times less cell material than in the tandem affinity purification approach (Elzi et al., 2014).

ZEB1 is a transcription factor mediating epithelial-to-mesenchymal transition during development, but also in tumor progression (Zhang et al., 2015). To identify potential co-repressors of ZEB1, BioID was employed and allowed the identification of every core member of the nucleosome remodeling and deacetylase (NuRD) complex (Manshouri et al., 2019). Of note, the authors fused BirA^∗^ to either the N- and C-terminus of ZEB1 and only considered candidates present in both proxisomes and not present in the control. With this approach, they could identify 68 potential interactors of ZEB1. Subsequent experiments revealed the Rab22 GTPase-activating protein TBC1D2b gene as a ZEB1/NuRD complex target. TBC1D2b is crucial for suppressing E-cadherin internalization, a process that promotes the epithelial-to-mesenchymal transition.

### Chromatin Remodelers and Topoisomerases

Chromatin remodelers play a key role in reshaping the chromatin landscape to grant access to transcription, replication or DNA-repair factors. They can either influence the DNA-binding properties of histones through N-terminal modifications or directly move, evict or restructure nucleosomes in an ATP-dependent manner. For example, the histone methyltransferase NSD2 specifically dimethylates histone H3 lysine 36 (H3K36me2). This modification is associated with gene activation and overexpression of NSD2 has been linked to some forms of cancer (Kuo et al., 2011). To uncover potential interaction partners of NSD2, a BirA^∗^ fusion protein was overexpressed in NSD2 stable knock-out cells (Huang et al., 2019). The authors pursued both a qualitative and quantitative approach. First, they analyzed six biological replicates of NSD2-BirA^∗^ expressing cells against wild type cells, resulting in 63 candidates. In the second approach, label-free quantitative mass spectrometry analysis of NSD2 BioID with NLS-BirA^∗^ as the control resulted in 24 nuclear candidates. The overlap between the qualitative and quantitative approaches provided 16 potential high-confidence interactors. Further characterization of hits identified PARP1 as a regulator of NSD2 upon DNA-damage.

Topoisomerases are necessary for all biological processes that require DNA topology changes, including transcription, replication or chromatin remodeling (Chen et al., 2013). Thus, protein-protein interaction maps of topoisomerases would be particularly important to understand their essential functions in the cell, but difficult to achieve due to their insoluble properties in biochemical assays. BioID with topoisomerase IIβ as bait and no BirA^∗^, GFP-BirA^∗^ or NLS-BirA^∗^ as controls could identify 25 proximal proteins, of which 4 were known and 21 unknown (Uusküla-Reimand et al., 2016). Here, the usage of three distinct kinds of controls increased the stringency of analysis. The authors could subsequently confirm TOPIIβ associations with CTCF and cohesin subunits at the boundaries of topologically associating domains.

### DNA Repair and Replication Factors

The MCM2-7 complex is known for its helicase activity during replication in S-phase, but has also been associated with DNA repair, chromatin organization and cell cycle regulation (Bailis and Forsburg, 2004). In an attempt to identify a more complete interaction map, Dubois et al. employed affinity or proximity purification followed by LC-MS/MS in a side-by-side comparison (Dubois et al., 2016). To this end, the authors fused either GFP or BirA^∗^ to each of the six MCM2-7 subunits and using the SILAC method subsequently pulled down with GFP nanobodies or streptavidin beads. The BioID approach generated roughly the same number of potential interactors as AP-MS. Interestingly, in this case the two approaches only shared ∼15–20% of candidate hits, but it is not clear if this could originate from high background of both methods due to the endogenously high expression level of MCM complexes in cells (Dubois et al., 2016). Following etoposide treatment, they could identify DNA damage specific MCM interactors including the DDB1-CUL4 complex involved in nucleotide excision repair. Unfortunately, no BirA^∗^ reference (e.g., NLS-BirA^∗^) was used in this study, increasing potential false-negatives. However, they could still generate high confidence hits due to very stringent cut-offs and to the improved statistical power of 12 data sets merged from the two purification approaches.

In a similar approach, the same group probed the interactome of the master regulator HNF4α, which plays a crucial role in development and tumorigenesis (Babeu et al., 2019). Increased expression of the isoform P2-HNF4α has recently been implicated in colorectal cancer. BioID with P2-HNF4α-BirA^∗^ and immunoprecipitation with P2-HNF4α-GFP in HEK293T or HCT116 (colorectal cancer cell line without HNF4α expression) revealed an association of P2-HNF4α with DNA repair factors including PARP1, RAD50, and PRKDC. They confirmed these interactions by co-immunoprecipitation with endogenous HNF4α in colorectal cancer cell lines. Here, BioID generated about four times more candidates than AP-MS, but also had a higher background. Interestingly, the DNA repair factors were found in the relatively small overlap of both approaches. This suggests that using both methods simultaneously can potentially provide biologically relevant candidate hits.

Another example of coupling proximity and affinity purification with mass spectrometry is a study investigating the interactome of the DNA repair factor Ku70 (Abbasi and Schild-Poulter, 2019). Besides its well-known role in non-homologous end-joining, Ku70 is also implicated in other chromatin processes, e.g., transcriptional regulation or DNA replication (Mo and Dynan, 2002; Abdelbaqi et al., 2013). BioID identified a total of 501 candidates across three biological replicates, while AP-MS detected 282. Interestingly, on average, ∼55% of BioID candidates of a biological replicate were present in all three biological replicates, whereas this proportion was only ∼18% for AP-MS. This indicates that the AP-MS method is not as reproducible as BioID for probing the interactome of Ku70.

Together, these studies indicate that proximity labeling is able to discover physical interactors that are also found in AP-MS experiments. However, the overlap during side-by-side experiments is usually small. Interestingly, ∼50% of candidates of the SOX2 BioID proxisome could be found in at least one of eight different SOX2 AP-MS interactomes (Kim et al., 2017). The individual overlaps of the AP-MS interactomes with the BioID proxisome range from ∼0 to 40% (Supplementary Table 5 in Kim et al., 2017). This indicates a large variation in the AP-MS interactomes and is most likely due to experimental design factors in the AP-MS experiments, e.g., cell type, crosslinking conditions, enrichment strategy and analysis parameters. It will be interesting to see if proximity labeling is less susceptible to variations resulting from different experimental designs. The three MYC BioID studies described above had a large overlap, but the experimental parameters were very similar (see section Transcription Factors and Figure 3A). When comparing two studies with Lamin A as bait in the same cell type, but with different enrichment and mass spectrometry analysis strategies, there is still a decent overlap of candidates (Figure 3B). Based on these examples, the studies suggest that the generated candidate lists of BioID proximity labeling experiments are less susceptible to variations caused by experimental design factors than AP-MS, but more comparative studies on distinct targets will be needed to verify this speculation.

### Locus Specific

All methods described until now give insight into chromatin interactions that can occur genome-wide without any spatial information. However, it would also be interesting to investigate protein-protein interactions at specific DNA loci, especially in the context of oncogenes. The most commonly used method is a special form of IP-MS called chromatin immunoprecipitation (ChIP). There are two types of ChIP: cross-linked (XChIP) and native ChIP (NChIP). In XChIP, the chromatin is reversibly cross-linked with associating proteins and subsequently sheared by sonication. For NChip, the native chromatin is extensively digested by micrococcal nuclease (MNase). To immunoprecipitate the local chromatin environment, antibodies targeting histone posttranslational modifications or chromatin factors of interest are used. The isolated and purified DNA is then sequenced to allocate genomic locations of the protein-protein or protein-DNA interaction. NChip is mostly limited to histone proteins due to their high abundance and stable interaction with DNA, whereas other proteins are lost without crosslinking during the stringent IP conditions. However, XChIP can generate false positives by crosslinking randomly associating proteins or after cell lysis by non-specific binding of factors to the sheared chromatin or bead material. Furthermore, crosslinking agents distort the native environment of chromatin before analysis (Beneke et al., 2012; Gavrilov et al., 2015).

Another method is the Proteomics of isolated chromatin segments (PICh), which deploys complementary DNA probes after chemical crosslinking to capture the local chromatin composition (Déjardin and Kingston, 2009). A different approach targets a specific genomic region with a site-specific recombinase that can then be purified by affinity purification (Griesenbeck et al., 2003; Hamperl et al., 2014). Recently, the CUT&RUN method was introduced as an alternative to ChIP for genome wide profiling of the local chromatin environment of a chromatin factor of interest (Skene and Henikoff, 2017). In this *in situ* approach, protein A-fused MNase is directed to a specific antibody against the chromatin target of interest and leads to the release of protein-DNA complexes into solution without the requirement of crosslinking agents (Skene and Henikoff, 2017). As this technique basically represents a proximity-based reaction in close to native conditions, it will be interesting to pursue how the CUT&RUN method could complement BioID and APEX studies.

The development of a catalytically dead dCas9-BirA^∗^ fusion protein has laid the foundation for an *in vivo* approach using proximity labeling (Schmidtmann et al., 2016). In principle, cells expressing dCas9-BirA^∗^ in combination with a single guide RNA can be targeted to any genomic locus of interest. Incubation with biotin should then allow to label the locus-proximal proteins *in vivo*. This original approach, termed CasID, was validated by targeting the repetitive sequences of telomeres, major satellite and minor satellite DNA (Schmidtmann et al., 2016). The authors could identify known interactors but also validated zinc-finger protein 512 as a new major satellite repeat associating protein. However, the generated telomere protein list was rather short, with only seven significantly enriched proteins. It was possible to increase BirA^∗^ activity and thereby gain more protein enrichment by designing a longer flexible glycine-serine linker between the dCas9 protein and BirA^∗^ (Li et al., 2019). In this study, they were able to generate a telomere associating protein list of over 300. Although increasing the chance for false positives with this extended linker approach, the authors could identify and validate desmoplakin as a telomere associating protein. To target single copy loci in the future, critical steps to optimize may include using multiple sgRNAs targeting the same locus, increasing cell numbers or optimizing the streptavidin pulldown (Schmidtmann et al., 2016).

The next advance tried to address the slow reaction dynamics of BirA^∗^ by fusing APEX2 to dCas9. Similar to the studies with dCas9-BirA^∗^, in this approach termed dCas9-APEX2 biotinylation at genomic elements by restricted spatial tagging (C-BERST), first the telomeres and centromeres were targeted, which allowed specific profiling of their subnuclear proteomes (Gao et al., 2018). Simultaneously, an approach to study non-repetitive single loci, termed genomic locus proteomics (GLoPro) was developed (Myers et al., 2018). The authors used five different sgRNAs targeting and tiling the same locus. These sgRNAs were expressed in separate HEK293T cell lines. Consequently, they were able to overlap the data sets and eliminate common noise. To limit artifacts from constitutive overexpression of dCas9-APEX2, expression was fine-tuned by an inducible promotor. With this approach, a snapshot of the proximal proteome of the *hTERT* and *c-MYC* promoters were obtained. In general, a benefit of these approaches is the possibility of using a simple and highly effective control without sgRNA or a non-specific sgRNA.

Another method was not only able to identify locus-specific proximal proteins, but also RNA and long range DNA-interactions by subsequent chemical crosslinking and high-throughput sequencing (Qiu et al., 2019). The authors did not fuse APEX2 directly to dCas9, but expressed a sgRNA that contains MS2 stem loops. This secondary structure is then specifically recognized by the MS2 coat protein (MCP) fused to APEX2. A major drawback of this approach could be non-bound MCP-APEX2 fusion proteins that generate false-positives. In agreement, the authors show that low expression of MCP-APEX2 is necessary for successful enrichment.

Protein-protein interactions at telomeres are of broad interest, because telomere length plays an important role in tumorigenesis. Telomerase is reactivated in most cancers, but there are cancers in which telomerase is suppressed and telomeres are maintained by alternative lengthening of telomeres (ALT). To identify proximal factors of ALT cell telomeres, BioID proximity labeling with TRF1-BirA^∗^ was used (Garcia-Exposito et al., 2016). By comparing the proxisome of ALT-positive U2OS with telomerase-positive HeLa cells, they were able to identify a role of translesion DNA synthesis in the ALT mechanism. Since biotin labeling occurs over all the different cell cycle states of telomeres, the BioID approach has the advantage over the previously used PiCH method (Déjardin and Kingston, 2009) to give a more comprehensive overview of protein interactions at telomeres. However, PiCH or APEX2 provide a “snapshot” and would therefore be superior when combined with cell synchronization if the goal is to analyze different time points during the cell cycle.

In summary, it is possible to use proximity labeling to identify the proxisomes of specific loci by employing dCas9 fusion proteins. However, targeting non-repetitive single loci is challenging, due to the low number of bound proximity labeling enzymes and resulting low biotinylation levels. In most studies, repetitive DNA was targeted greatly increasing the signal-to-noise ratio. Nevertheless, this limitation could be partially overcome by tiling the locus with multiple sgRNAs, as performed with the *hTERT* and *c-MYC* promoters (Myers et al., 2018). Thus, it could become feasible that the proxisomes are determined even at single copy gene loci, but that remains to be seen.

### Nuclear Compartments

Instead of focusing on specific factors or genomic loci, another emerging application of proximity labeling is the analysis of whole compartments or difficult to isolate/purify components of the nucleus, such as the nuclear envelope, centromeres, or nuclear bodies. As a proof-of-principle, the nuclear lamina was targeted by BioID of Lamin A, a major component of this nuclear compartment (Roux et al., 2012). Lamin A was also studied in the context of Hutchinson-Gilford progeria, a premature aging syndrome. Here, BirA^∗^ was fused to normal Lamin A or the truncated form characteristic to this disease, called progerin (Chojnowski et al., 2015). By comparing the differential abundance of proximal proteins, they could detect reduced association of LAP2α with progerin compared to Lamin A. In another approach, the proxisome of Lamin B1 was explored with a lenti-virus-delivered LMNB1-BirA^∗^ fusion protein (Fu et al., 2015). This mode of delivery to perform BioID may be of advantage in cells that are difficult to transfect. A different study tried to address some key issues of the BioID approach while probing the proxisome of LAP2β, another inner nuclear membrane component. The conventional fusion protein LAP2β-BirA^∗^ is too large for correct localization, because it cannot travel through the nuclear pore complex from the outer to the inner nuclear membrane. To circumvent this restriction, a method with the rapamycin inducible dimerization between FK506 binding protein (FKBP) and FKBP-rapamycin binding (FRB) combined with BioID was developed (Chojnowski et al., 2018). In short, FRB (∼10 kDa) was fused to LAP2β and FKBP to BirA^∗^. This smaller fusion protein was able to pass the nuclear pore complex and localize correctly. Subsequently, the rapamycin induced dimerization allowed FKBP-BirA^∗^ to bind FRB-LAP2β. Importantly, this system is internally controlled without addition of rapamycin and seems to reduce false-positive identifications (Chojnowski et al., 2018). However, false-positives are still conceivable when the dimerization occurs in the cytoplasm before LAP2β is relocated to the inner nuclear membrane. In a similar approach, the nuclear vicinity of vesicle-associated membrane protein-associated protein B (VABP) was explored with rapamycin directed APEX2 (James et al., 2019). VABP localizes primarily to the ER, but also to the inner nuclear membrane. In this study, the APEX2-FKBP fusion protein was additionally tagged with a nuclear localization signal. In combination with FRB-VABP, this allowed the specific enrichment of the nuclear proxisome of VABP.

Besides the nuclear lamina, certain nuclear bodies were analyzed by proximity labeling. SUP-46 is a *Caenorhabditis elegans* RNA binding protein with an essential role in sustaining transgenerational germline immortality. Proxisome analysis of the human homologs MYEF2 and HNRNPM with BioID revealed robust associations with paraspeckles, nuclear stress granules and the nucleolus (Johnston et al., 2017). Interestingly, a large overlap of ∼60% was observed among the 133 and 110 candidates in the MYEF2 and HRNPM BioID assays, respectively.

## Conclusion and Perspectives

Recent developments in proximity labeling techniques have provided a valuable platform to study chromatin in new ways. BioID, APEX and their successors have become a valuable complementation to classical nuclear protein-protein interaction studies like AP/MS and ChIP. Different variations of these assays have started to shed light on the native environment of specific chromatin factors, specific gene loci or even whole nuclear compartments.

Interestingly, peroxidases can also directly label RNA and potentially DNA with biotin (Fazal et al., 2019; Zhou et al., 2019). With APEX-seq, it is possible to probe the vicinity not only for proteins, but also for various forms of RNAs. It has been used in parallel to APEX-MS to study the organization of the translation initiation complex and repressive RNA granules (Padrón et al., 2019). In the future, this approach can potentially uncover the localization of RNAs, e.g., lncRNAs, in the native vicinity of proteins or specific loci.

Proximity labeling also has the capability of studying microprotein-protein interactions (Chu et al., 2017). Small open reading frames (smORFs) encode hundreds of thousands of microproteins and small peptides, of which only few have been characterized. However, some of these microproteins have important biological functions and uncovering their native context in the cell can give clues regarding function.

Recently, combinations of proximity labeling and protein-fragment complementation assays (PCA) were developed. In a PCA, two POI are fused to either half of a split reporter protein (enzyme or fluorescent protein). The reporter protein is reconstituted only upon interaction of the POI. However, the exact interaction dynamics between the two split fragments remain unknown, e.g., if the reconstitution is reversible. For proximity labeling, split-BioID and split-APEX2 have now been reported (Munter et al., 2017; Schopp et al., 2017; Xue et al., 2017; Han et al., 2019). As the biotinylation is dependent on the correct localization of both targeted factors, this approach can significantly reduce the number of false positives (Munter et al., 2017). This approach is specifically interesting for transient protein interactions, where labeling only occurs at the right time and the right site when a protein complex is formed or a biological process has been initiated. Furthermore, splitting the reporter enzyme results in smaller tags for the POI and therefore potentially less functional impact.

A recent paper indicated that biotin ligase-based proximity labeling may potentially allow the study of intrinsically unstructured regions. These flexible lysine-rich protein domains are more accessible and show faster biotinylation kinetics than structured, less exposed regions (Minde et al., 2020). Consequently, a time course biotin “painting” approach could even give insight into differences of secondary or tertiary protein structures. In conclusion, proximity labeling is emerging as a powerful complementary tool to study the local environment of chromatin factors that can significantly improve our understanding of the complex interaction networks in the nucleus.

## Author Contributions

HU searched the literature, created the figures, and wrote and edited the manuscript. SH provided the guidance and edited the manuscript.

## Conflict of Interest

The authors declare that the research was conducted in the absence of any commercial or financial relationships that could be construed as a potential conflict of interest.
